# Supplementary material for: The microbiome biomarkers of pregnant women’s vaginal area predict preterm prelabor rupture in Western China
Source: Front Cell Infect Microbiol. 2024 Oct 31;14:1471027. doi: 10.3389/fcimb.2024.1471027 (PMC11560878; doi:10.3389/fcimb.2024.1471027)
Supplement: Supplementary file 1 [file DataSheet1.zip › compare_1/Community/Pieplot/genus/sample/C22.genus.pieplot.pdf]

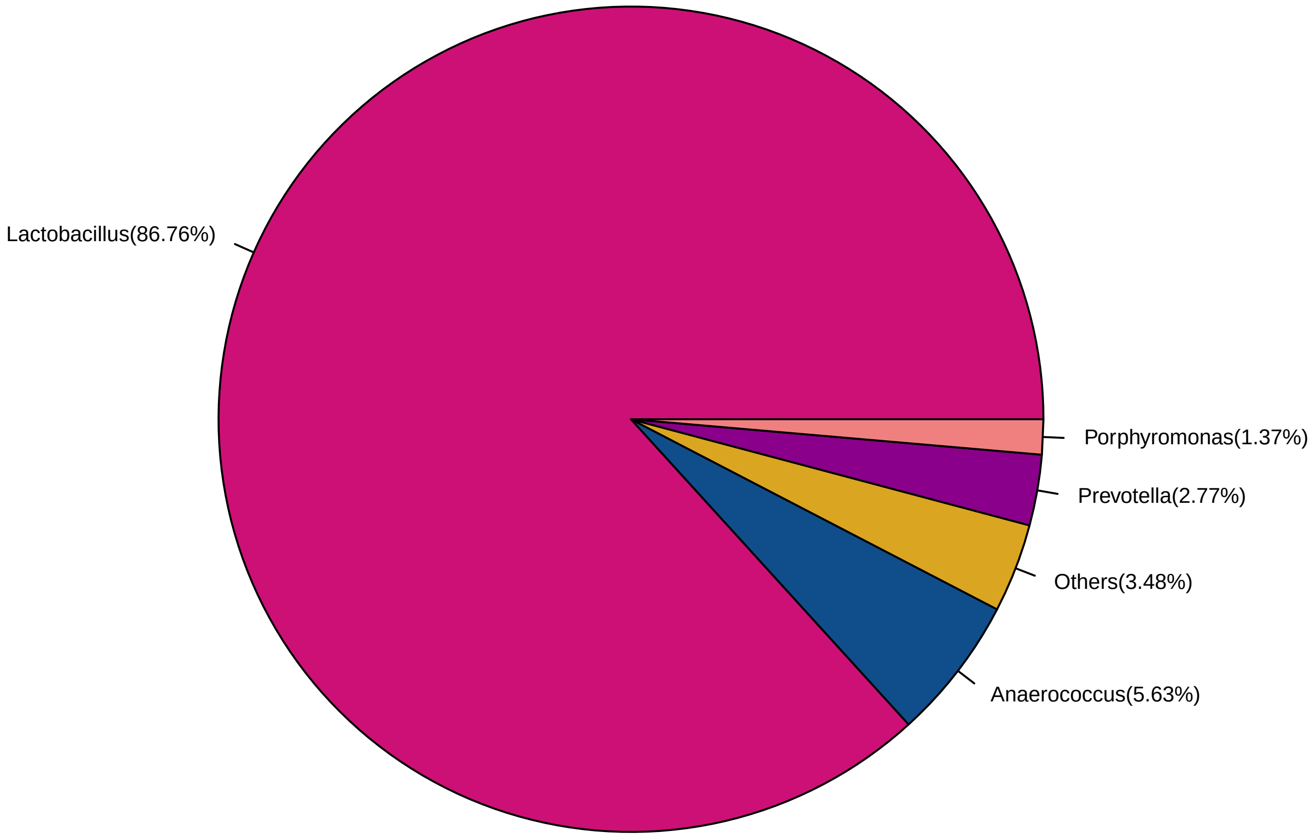
Lactobacillus(86.76%)
Porphyromonas(1.37%)
Prevotella(2.77%)
Others(3.48%)
Anaerococcus(5.63%)
